# Feasibility and Safely of Oral Rehydration Therapy before Upper Gastrointestinal Endoscopic Submucosal Dissection

**DOI:** 10.1155/2020/4372503

**Published:** 2020-08-06

**Authors:** Yasutoshi Shiratori, Takashi Ikeya, Kenji Nakamura, Katsuyuki Fukuda

**Affiliations:** ^1^Department of Gastroenterology, St. Luke's International Hospital, Tokyo 104-8560, Japan; ^2^Department of Gastroenterology, Tokyo Dental University Ichikawa General Hospital, Chiba 272-8513, Japan

## Abstract

**Methods:**

We used alginade water (125 mL, Nestle Co., Kobe, Japan) for PORT. Alginade water is a flavored sports drink, which is palatable and promotes wound healing due to a high concentration of alginate. We conducted a single-center single-arm prospective feasibility study of PORT in 244 patients who underwent upper gastrointestinal ESD. The group wherein PORT was administered up to two hours before ESD (*n* = 120) was compared with the historical control group (non-PORT group, *n* = 120). We investigated the total fluid intake, hematocrit change, complications due to PORT, complications during ESD, ESD procedure time, and length of hospital stay in each group.

**Results:**

The average fluid intake in the PORT group was 462.6 mL. No complications were observed due to PORT and ESD, and significant differences in the ESD procedure time or hospital stay was not noted.

**Conclusion:**

PORT up to two hours before upper gastrointestinal ESD is feasible.

## 1. Introduction

Fasting is commonly noted before upper gastrointestinal endoscopic treatment. Patients experience physical stress, such as thirst, hunger, and mental restraint, before endoscopic treatment. The fasting period is set at each hospital, and there are no established guidelines.

Preoperative oral rehydration therapy (PORT) is commonly performed before surgery. Regarding the fasting period before surgery, societies of anesthesiology in the United States and most European countries recommend the intake of clear fluids up to two hours before surgery [1–3]. In contrast, PORT is not commonly performed in the endoscopic field. There are a few reports on the use of PORT before upper gastrointestinal endoscopy; therefore, the feasibility and usefulness of PORT in such settings are unclear. This study is aimed at evaluating the feasibility of PORT before upper gastrointestinal endoscopic submucosal dissection (ESD).

## 2. Materials and Methods

**Figure 1:**
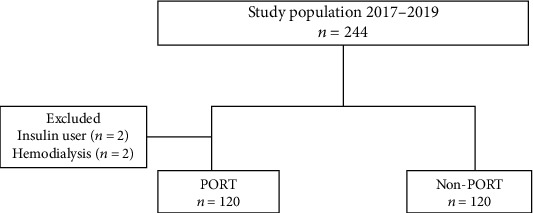
Study flow diagram showing the selection of cases. For a total of 244 patients, upper gastrointestinal endoscopic submucosal dissection was performed in a tertiary hospital in Tokyo, Japan. Of these, 240 patients were included in this study. The patients were classified into the PORT and non-PORT groups. PORT: per oral rehydration therapy.

Upper gastrointestinal ESD was performed in 244 patients at our hospital between April 2017 and December 2019. We conducted a single-arm prospective feasibility study of PORT compared with historical control (non-PORT group). As shown in Figure 1, patients were classified into two groups, as follows: 120 patients received PORT, and 120 patients did not receive PORT. ESD without PORT was performed from April 2017 to September 2018, whereas ESD with PORT was performed from October 2018 to December 2019. Patients with difficulty in swallowing, those with diabetes requiring insulin, those with severe heart failure or hemodialysis, and those who did not provide consent were excluded (in total, four patients were excluded from this study; two patients were excluded because of insulin use and two patients were excluded as they underwent hemodialysis). This study was approved by our Institutional Review Board (18-R149, June 31, 2018).

**Figure 2:**
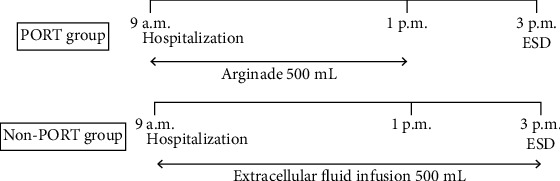
Time course for the PORT group and non-PORT group. In the PORT group, 500 mL of alginade water was administered up to 2 hours before ESD. In contrast, 500 mL of extracellular fluid infusion was administered in the non-PORT group before ESD. ESD: endoscopic submucosal dissection; PORT: preoperative oral rehydration therapy.

We used alginade water (125 mL, Nestle Co., Kobe, Japan) for PORT. Alginade water is a flavored sports drink, which is palatable and promotes wound healing due to a high concentration of alginate. Alginade water is 1.3 dollars per pack, and the cost of 500 mL (5.2 dollars) was paid for each person at our hospital. The composition of alginade water is shown in Table 1. In both groups, patients were instructed to eat by 8 p.m. (the day before ESD) and to drink clear water until the time of admission (9 a.m.). In the PORT group, 500 mL of alginade water was administered up to two hours before ESD, and an extracellular fluid infusion was administered just before ESD. On the contrary, clear water (until the time of hospitalization) and 500 mL of extracellular fluid infusion were administered before ESD in the non-PORT group.  Figure 2 illustrates the time course of PORT, assuming the start of ESD at 3 p.m. If ESD is initiated earlier or later than 3 p.m., the patient is allowed to drink alginade until two hours before the commencement of ESD. ESD was performed with conscious sedation using midazolam and pethidine hydrochloride.

We investigated the total fluid intake, extracellular fluid infusion, complications due to PORT, complications during ESD, and hematocrit before admission and on the morning after ESD in the PORT and non-PORT groups. The total fluid intake was assessed by the nurse in charge by measuring the weight of the alginade water pack. Complications due to PORT were defined as symptoms, such as nausea and vomiting, aspiration, cardiopulmonary events, increased blood sugar levels requiring insulin treatment, and abnormal electrolyte levels. Complications during ESD were defined as perforation and aspiration pneumonia. Similarly, we compared the procedure time of ESD and length of hospital stay in both groups. Procedure time was defined as the time from the commencement of ESD (marking of the lesion) to the completion of ESD (en bloc resection). Statistical analysis was performed using Stata version 16 (Stata Corp. USA). Student's *t*-test and Fisher's exact test were used for the continuous and categorical variables, respectively.

## 3. Results

After applying the exclusion criteria, a total of 240 patients were categorized into two groups (120 with PORT, 120 without PORT). No significant differences were observed in patient characteristics between the two groups (Table 2). The average volume of the oral rehydration solution in the PORT group was 462.6 mL. None of the patients suffered from complications due to PORT. In addition, no statistical differences were observed in hematocrit, procedure time in ESD, or duration of hospitalization (Table 3).

## 4. Discussion

This study investigated the feasibility and safely of administering PORT up to two hours before upper gastrointestinal ESD. ESD was performed safely, without any complications. The results suggest the feasibility of PORT before ESD.

The guidelines of the American Society for Gastrointestinal Endoscopy do not provide clear recommendations on the duration of fasting. Previously, clear water could be consumed up to four hours prior to upper gastrointestinal endoscopy [4]. However, it has recently been reported that endoscopy is not affected by a shorter fasting time, such as one or two hours [5].

The usefulness of PORT has been previously reported in the field of surgery and anesthesiology. Randomized controlled trials have shown that clear fluid ingestion up to two hours prior to surgery did not affect gastric remnant fluid or gastrointestinal surgery [6, 7]. In this study, PORT up to 2 hours before ESD treatment was similarly feasible. At our institution, ESD mainly starts between 2 p.m. and 4 p.m.; however, it may be possible to increase the amount of PORT at other hospitals with a later ESD start time. Similarly, in hospitals where ESD is performed in the morning, patient satisfaction may be improved by providing PORT from the previous evening's dinner or later until two hours before treatment. Further, studies on gastric volume using magnetic resonance imaging have shown that both healthy and morbidly obese people excrete their stomach contents two hours after eating and return to their fasting state [8, 9]. A study on gastric contents using ultrasonography has revealed the safety of oral intake up to two hours prior to general anesthesia [10]. In addition, another PORT study reported a reduction in the volume of infusions administered and the burden on the nurses associated with patients [11, 12]. In this study, although a detailed measurement of the remaining gastric juice could not be performed because there was almost no residue in the course of performing ESD. Even if there was a residue, it supposedly resulted from drinking the pharyngeal anesthetic liquid, and only a short endoscopic suction was necessary to remove the gastric juice.

Regarding the type of solution used for PORT, there are observational studies that OS-1 (Otsuka Co., Japan) and alginade water do not differ in terms of intake volume, patient satisfaction, or laboratory measurements such as electrolytes [13]. In a trial wherein 250 mL of alginade water was drunk up to two hours prior to an abdominal operation, the product was confirmed to be safe and effective [14].

In this study, the patients were aged 70 years on average. Especially in the elderly, fasting before therapeutic endoscopy can increase the risk of dehydration [15]. PORT may aid in preventing dehydration. Furthermore, the present study revealed the safety of oral intake up to two hours before upper gastrointestinal endoscopy. The results may contribute to not only before upper gastrointestinal ESD but also to the fields of daily upper gastrointestinal endoscopy. Further studies are needed to determine whether PORT can similarly be utilized prior to conventional upper gastrointestinal endoscopy.

There are several limitations to this study. First, this is an observational study from a single center. Increasing the study size across multiple centers would improve the meaningfulness of the data. Second, the amount of alginade water consumed varied between individuals. Third, whether PORT can be safely performed in very elderly patients, namely, those aged >80 years.

## 5. Conclusions

This study indicates that PORT up to two hours before ESD is feasible and safe. The results presented in this study are encouraging and warrant further prospective trials involving larger numbers of patients.

## Figures and Tables

**Table 1:** Composition of the oral rehydration solution.

Alginate water (per pack)	
Volume (mL)	125
Energy (kcal)	100
Carbohydrate (g)	22.5
Protein (g)	5 (alginic acid 2.5)
Sodium (mg)	0
Potassium (mg)	400
Vitamin A (*μ*g)	150
Vitamin C (mg)	500
Vitamin E (mg)	5
Zinc (mg)	10
Iron (mg)	0

**Table 2:** Patient characteristics.

	PORT (120)	Non-PORT (120)	*P* value
Age (mean ± SD)	70.6 ± 11.7	71.9 ± 10.4	0.47
Sex (male, female)	93, 27	90, 30	0.71
Hematocrit before admission (mean ± SD, %)	36.9 ± 3.7	37.4 ± 3.8	0.85
Comorbidity for Charlson's score (mean ± SD)	1.0 ± 1.7	1.1 ± 1.5	0.57
Myocardial infarction	5	6	0.76
Cerebral infarction	3	3	1
Diabetic mellitus	10	12	0.66
Chronic kidney disease	4	5	0.73
Antithrombotic therapy	8	10	0.62
Location (esophagus, stomach, and duodenum)	39, 79, 2	44, 76, 0	0.48

PORT: preoperative oral rehydration therapy; SD: standard deviation.

**Table 3:** Outcomes of the PORT group and non-PORT group.

	PORT	Non-PORT	P-value
Total intake (mean ± SD, mL)	462.6 ± 29.2	0	N/A
Volume infusion	0	500	N/A
Complications due to PORT	0	N/A	N/A
Complications during ESD	0	0	N/A
Hematocrit change (mean ± SD, %)	1.3 ± 1.7	1.4 ± 1.4	0.75 (95% CI: -0.5, 0.3)
Procedure time (mean ± SD, min)	73.2 ± 4.8	77.9 ± 4.9	0.72 (95% CI: -15.8, 22.8)
Hospitalization days (mean ± SD)	5.2 ± 0.1	5.2 ± 0.1	0.93 (95% CI: -0.16, 0.18)

CI: confidence interval; ESD: endoscopic submucosal dissection; N/A: not applicable PORT: preoperative oral rehydration therapy; SD: standard deviation.

## Data Availability

The procedure data used to support the findings of this study are included within the article.
